# Identification of psychological features and development of an assessment tool for event-related psychological distress after experiencing non-traumatic stressful events

**DOI:** 10.1371/journal.pone.0249126

**Published:** 2021-03-31

**Authors:** Ryota Seki, Tasuku Hashimoto, Mami Tanaka, Hiroki Ishii, Michi Ogawa, Aiko Sato, Atsushi Kimura, Akihiro Shiina, Michiko Nakazato, Masaomi Iyo

**Affiliations:** 1 Department of Psychiatry, Graduate School of Medicine, Chiba University, Chiba, Japan; 2 Department of Psychiatry, School of Medicine, International University of Health and Welfare, Chiba, Japan; 3 Division of Clinical Study on Juvenile Delinquency, Center for Forensic Mental Health, Chiba University, Chiba, Japan; 4 Division of Medical Treatment and Rehabilitation, Center for Forensic Mental Health, Chiba University, Chiba, Japan; Department of Psychiatry and Neuropsychology, Maastricht University Medical Center, NETHERLANDS

## Abstract

Stressful events in daily life that are non-traumatic (e.g., family-, school-, work-, interpersonal-, and health-related problems) frequently cause various mood disturbances. For some people, being exposed to non-traumatic but stressful events could trigger the onset and relapse of mood disorders. Furthermore, non-traumatic stressful events also cause event-related psychological distress (ERPD), similar to that of post-traumatic stress disorder (PTSD; i.e., intense intrusive imagery or memory recall, avoidance, and hyperarousal) in the general population and individuals with mood disorders. However, previous ERPD studies only showed that people with ERPD display PTSD-like symptoms after non-traumatic experiences; they failed to get to the crux of the matter by only utilizing trauma- or PTSD-related assessment tools. We thus aimed to identify the psychological phenomena and features of ERPD after individuals experienced non-traumatic stressful events, and to develop and validate an appropriate ERPD assessment tool. First, we conducted a qualitative study to obtain the psychological features through interviews with 22 individuals (mean age = 41.50 years old, *SD* = 12.24) with major depressive disorder or bipolar disorder. Second, in the quantitative component, we implemented a web-based survey with 747 participants of the general population (mean age = 41.96 years old, *SD* = 12.64) by using ERPD-related questionnaires created based on the qualitative study; then, we examined the reliability and validity of the ERPD assessment tool. Results yielded that the psychological features of ERPD comprised four factors: feelings of revenge, rumination, self-denial, and mental paralysis. These were utilized in the developed 24-item measure of ERPD—a novel self-report assessment tool. For various professionals involved in mental healthcare, this tool can be used to clarify and assess psychological phenomena in people with ERPD.

## Introduction

Stressful events in daily life that are non-traumatic (e.g., family-, school-, work-, interpersonal-, and health-related problems) frequently cause various mood disturbances, such as depressed mood and anger, in people of all ages [1–4]. For some people, after exposure to non-traumatic life events, which are less serious than traumatic ones (e.g., near-fatal accidents, war, child abuse, and serious violence), they could have severe mood disturbances; consequently, non-traumatic events could trigger the onset and relapse of mood disorders including major depressive [4–6] and bipolar [5, 7, 8] disorders. Furthermore, non-traumatic stressful events also cause event-related psychological distress (ERPD), similar to that of post-traumatic stress disorder (PTSD; i.e., intense intrusive imagery or memory recall, avoidance, and hyperarousal) in the general population [9, 10]. Moreover, prior ERPD studies demonstrated that almost patients with major depressive [11] or bipolar [12] disorder experienced ERPD after non-traumatic stressful events, unremitted patients have more severe ERPD than do remitted patients, and the severity of ERPD is positively correlated with depressive symptoms in patients with those two disorders. Given that non-traumatic life events can happen to everyone, investigating the psychological features and assessment method of ERPD is important to understand and treat people with ERPD.

Regarding previous studies about ERPD, there is a consistent and essential limitation—researchers utilized trauma- or PTSD-related assessment tools (i.e., the Trauma Memory Quality Questionnaire [13], the Post-traumatic Stress Symptom Scale–Self-report version [14], or the Impact of Event Scale–Revised (IES-R)) [15, 16]. These were originally developed for people who had traumatic experiences and/or were diagnosed with PTSD. Hence, prior ERPD studies only showed that people with ERPD after non-traumatic experiences display PTSD-like symptoms; they fail to get to the crux of the matter of ERPD. Furthermore, the differences between the psychological features of ERPD after experiencing non-traumatic stressful events and those of PTSD are unknown.

The aims of this study were to identify the psychological phenomena and features of ERPD after individuals experienced non-traumatic stressful events, and to develop and validate an appropriate ERPD assessment tool. First, we conducted a qualitative study to determine the psychological features of ERPD through interviews with individuals with major depressive disorder or bipolar disorder. Notably, all participants in in our previous studies stated that they had ERPD associated with their past non-traumatic events [11, 12]. Second, in a quantitative study, we implemented a web-based survey with the general population by using ERPD-related questionnaires created based on the qualitative study; then, we examined the reliability and validity of the ERPD assessment tool.

## Materials and methods

### Overall design

This study was conducted in two steps. The first step consisted of a qualitative study through semi-structured interviews with patients with mood disorders, to obtain the psychological features of ERPD associated with non-traumatic but distressing events, and to create initial assessment items. In the second step, we implemented a cross-sectional study with a web-based survey for the general population using ERPD-related questionnaires—created based on the first step data—then, we conducted an exploratory factor analysis to develop and validate the assessment tool of ERPD.

### Step 1: Qualitative research

#### Participants

Semi-structured interviews were conducted to collect the clinical features of ERPD in patients with major depressive disorder or bipolar disorder. The interviews were conducted at Chiba University Hospital, Sodegaura Satsukidai Hospital, Chiba Hospital, Kokoronokenkou Clinic Tsudanuma from September 2019 to March 2020. The qualitative research sample included 22 inpatients or outpatients (aged 20–65 years) who met the diagnostic criteria (Diagnostic and Statistical Manual of Mental Disorders-5; DSM-5) [17] after completing the Japanese version of the Mini International Neuropsychiatric Interview [18, 19]. Participants were all Japanese: 12 were recruited from Chiba University Hospital, four from Sodegaura Satsukidai Hospital, three from Chiba Hospital, and three from Kokoronokenkou Clinic Tsudanuma. We excluded patients who had dementia, organic mental disorder, schizophrenia, intellectual disability, or imminent suicidal ideation. Furthermore, we excluded patients with experiences of any traumatic events that met Criteria A for PTSD in the DSM-5, including complex PTSD as abuse and torture. In addition, we also excluded individuals with events associated with the novel coronavirus disease 2019 (COVID-19) since COVID-19 can cause various harms including increased mortality and critical medical conditions [20].

#### Semi-structured interview and procedure

In the semi-structured interview, one expert psychiatrist (RS) asked the patients with mood disorders about the information associated with ERPD. First, participants with no experience of traumatic events checked the corresponding items about events they had experienced in the non-traumatic event checklist, which classified stressful life events into 10 groups: family relationships, separation from a close person, interpersonal relationships, health issues, economic problems, anything to do with sexuality, changes in living conditions, a problem at work, bullying, and others. We also evaluated participants’ demographic characteristics, such as age, sex, and education level. Next, participants were instructed to describe the events they still feel most distressed about and recall most frequently. Then, participants further indicated 1) what happened (e.g., how old they were, what was the situation, when it happened, how long it lasted, if anyone helped them, etc.), 2) what they think about when recalling it, 3) how recalling it makes them feel, and 4) what their physical reactions were when recalling it.

### Step 2: Quantitative research

#### Participants

To conduct the web-based survey, we signed an agreement with Cross Marketing Inc.—a marketing company in Japan—which recruited participants over the Internet. The web-based survey applied The Checklist for Reporting Results of Internet E-Surveys [21]. In the web-based survey to verify the reliability and validity of the initial ERPD scale, 14 802 (89.1%) participants were excluded because they had not experienced ERPD, whereas 1817 people (10.9%) who had experienced ERPD more than one month prior to the survey were included. Of 1000 participants who completed the web-based survey, 253 participants were excluded for the following reasons: 97 participants stated events that were difficult to distinguish from trauma, such as severe accidents, child abuse, and disasters; 37 participants stated events associated with COVID-19; and 119 participants stated unspecified events. Thus, we analyzed data from 747 participants (mean age = 41.2 ± 12.6 years; 374 females and 373 males).

#### Measures

To measure ERPD, we used the 24 items created in Step 1. This scale—the ERPD-24—was a self-report assessment tool, scored with a four-point Likert scale: 0 (*not at all*) to 3 (*very much so*).

The IES-R is a self-report questionnaire to measure traumatic distress from traumatic events, which consists of three factors: intrusion (8 items), avoidance (8 items), and hyperarousal (6 items) [15]. It employs a five-point scale from 0 (*not at all*) to 4 (*extremely*). In the present study, values of Cronbach’s alpha were .93 for total IES-R score, .88 for intrusion, .84 for avoidance, and .82 for hyperarousal.

The Quick Inventory of Depressive Symptomatology (QIDS-J) [22, 23] was created to help providers screen for depression. It consists of a 16-item self-report scale and a four-point scale that measures sleep and mood, weight, concentration, guilt, suicidal ideation, interests, fatigue, and psychomotor changes that can be used to assess the severity of depression or screen for depression. In the present study, The Cronbach’s alpha coefficient of the QIDS-J ranged 0.68–0.73.

### Statistical analysis

We conducted an exploratory factor analysis for item selection and factor structure determination. Criteria for the number of dimensions included eigenvalues greater than 1.0, pattern of loadings in the dimensions, and interpretability of the solution. To test validity, we examined the correlations and partial correlations between the ERPD and IES-R, adjusted for sex, the period from event occurrence, and QIDS-J scores. Cronbach’s alpha coefficients were conducted to determine internal consistency. All analyses were performed using SPSS version 26 for Windows (IBM Corp., Armonk, NY, USA). Significance was set at *p* < .05.

### Ethics statement

The present protocol was approved by the ethics committee of Chiba University Graduate School of Medicine (ID 3456), Sodegaura Satsukidai Hospital, and Chiba Hospital, and Kokoronokenkou Clinic Tsudanuma. Before conducting semi-structured interviews, all patients provided informed written consent after the procedure was fully explained to them. Before the web-based survey, all participants received a brief description of the research and conditions for participation.

## Results

### Step 1: Classification of life events and item development process

Table 1 shows the characteristics of the 22 participants (11 males and 11 females, mean age = 41.50 years old, SD = 12.24), including 12 patients with bipolar disorder (six bipolar I and six bipolar II) and 10 patients with major depressive disorder. Among the 12 patients with bipolar disorder, seven patients were in a depressive state, three were in a hypomanic state, and two were in a euthymic state. Of the 10 patients with major depressive disorder, six were in a depressive state and four were in a euthymic state. Regarding the most stressful life events, separation from a close person was the most commonly occurring in patients with bipolar disorder, followed by interpersonal relationships; while interpersonal relationships was the most commonly occurring in major depressive disorder, followed by family relationships, health issues, or a problem at work.

**Table 1:** Participants’ characteristics in Step 1.

		Bipolar disorder (*n* = 12)	Major depressive disorder (*n* = 10)
Age, mean (*SD*)		39.9 (12.4)	43.4 (12.4)
Sex, *n*			
	Female	6	5
	Male	6	5
Highest level of school completed, *n* (%)			
	High school	3	4
	Vocational school	1	0
	Junior college	1	1
	University	7	5
Type of the most stressful life event, *n*			
	Family relationships	2	2
	Separation from a close person	4	1
	Interpersonal relationships	3	3
	Health issues	0	2
	Economic problems	1	0
	Changes in living conditions	1	0
	A problem at work	1	2

Table 2 shows the content analysis of the semi-structured interviews. First, we collected and organized similar items concerning participants’ thoughts, feelings, and physical reactions. We classified items into three categories—cognition, emotions, and physical reactions—using the extracted data. Next, with the help of a panel of expert psychiatrists (RS, TH, HI, MO, Aiko S, and AK) and one expert psychologist (MT), we created a draft of the ERPD-24, which were to be answered on a four-point scale. Higher total scores indicated higher ERPD.

**Table 2:** Item development and content analysis based on semi-structured interviews with participants.

Category during the recall	Response example	Scale item example
Cognitions	Remembering that the pressures of post-promotional work have crushed me, I blame myself	I blame myself when I recall the event
	I feel sorry for bothering the kids, thinking only of myself	I feel sorry for the people around me (e.g., family members, acquaintances, work colleagues, and/or classmates) when I recall the event
	I hate my divorced husband every time I remember	I feel hatred when I recall the cause of the event
	Recalling the repeated reprimands from management for three years, I want to get back at her	I feel like getting even when I recall the event
	I try not to remind myself that my roommates in the group home didn’t understand my illness, but I still remember	I cannot stop thinking about various scenes from the event
Emotions	Recalling being depressed by a cancer drug, I get scared	I feel fearful when I recall the event
	I feel unworthy when I remember that I didn’t do the work I needed to do, even though I worked both at home and on my days off	I feel as though I’m a worthless person when I recall the event
	I’m depressed when I recall my husband’s infidelity	I feel depressed when I recall the event
	I get annoyed when I remember that when I was little, my mother only loved my sister	I feel irritated when I recall the event
Physical reactions	Remembering that I left my job because of harassment from my boss makes my chest painful	I feel heaviness in my chest when I recall the event
	Recalling a bad relationship with my husband of more than 10 years makes it hard to breathe	I feel as if I’m suffocating when I recall the event
	Betrayed by someone I trusted, my body becomes sluggish	My body feels heavy when I recall the event
	Remembering my wife’s death from pancreatic cancer makes me lightheaded	My head feels foggy when I recall the event

### Step 2: Factor and reliability analysis of ERPD

To develop the ERPD-24, we performed an exploratory factor analysis. The items were subjected to a maximum likelihood factor analysis with direct promax rotation. We determined four factors with eigenvalues greater than one by examination of a scree plot. The first to fourth eigenvalues were 8.43, 3.37, 1.31, and 1.11, respectively, and the proportion of variance accounted for by the factor analysis was 51.52%. All items in each factor have factor loadings over .35 and there are no cross-loadings > .30. The first factor, “revenge,” was defined by seven items. The second factor, “rumination,” was defined by seven items. The third factor, “self-denial,” was defined by six items. The fourth factor, “mental paralysis,” was defined by four items (Table 3). The scale in its final version had very good internal consistency: Cronbach’s alpha was 0.91 for the complete scale and ranged from .82 to .88 for the four subscales.

**Table 3:** Exploratory factor analyses of the 24-item event-related psychological distress scale (*N* = 747).

		*Mean*	*SD*		Factor 1	Factor 2	Factor 3	Factor 4
Factor 1: Feelings of revenge (*α* = 0.88)								
	I feel angry when I recall the event	1.79	1.12		**.94**	-.08	-.05	-.08
	I feel irritated when I recall the event	1.70	1.09		**.81**	-.07	-.05	-.03
	I feel like getting even when I recall the event	1.28	1.16		**.79**	-.07	-.06	.03
	I feel hatred when I recall the cause of the event	1.63	1.09		**.74**	.05	.05	-.05
	I wish that the person who caused the event, or the cause itself, did not exist	1.93	1.10		**.61**	.05	-.01	-.02
	I feel sick when I recall the event	1.73	1.04		**.52**	.11	-.06	.19
	I feel that the event has ruined my life	1.18	1.06		**.47**	-.06	.18	.25
Factor 2: Rumination (*α* = 0.85)								
	I feel heaviness in my chest when I recall the event	1.21	1.02		-.18	**.81**	-.03	.14
	I feel as if I’m suffocating when I recall the event	1.37	.99		-.07	**.77**	-.04	.02
	I feel sad when I recall the event	1.62	1.06		-.08	**.77**	.03	-.09
	I feel depressed when I recall the event	1.73	1.02		.17	**.54**	.10	-.01
	I cannot stop thinking about various scenes from the event	1.74	.93		.26	**.48**	.08	.00
	I feel fearful when I recall the event	1.03	1.02		.01	**.43**	-.05	.18
	Even if I do not want to think about the event, I cannot stop myself	1.46	.95		.13	**.36**	.10	.16
Factor 3: Self-denial (*α* = 0.82)								
	I blame myself when I recall the event	.87	.96		-.18	.02	**.79**	.03
	I feel sorry for the people around me (e.g., family members, acquaintances, work colleagues, and/or classmates) when I recall the event	.90	.95		-.11	-.14	**.70**	.09
	I feel regret when I recall the event	1.29	1.05		-.02	.08	**.65**	-.09
	I do not believe that I handled the event well	1.65	1.05		.16	.11	**.60**	-.17
	I feel as though I’m a worthless person when I recall the event	.96	1.01		.02	-.09	**.56**	.28
	I feel miserable when I recall the event	1.50	1.02		.14	.20	**.51**	-.06
Factor 4: Mental paralysis (*α* = 0.85)								
	My body feels heavy when I recall the event	.82	.86		-.02	.08	-.09	**.80**
	I lose my will to do anything when I recall the event	.98	.96		.07	.02	-.04	**.78**
	My head feels foggy when I recall the event	.78	.85		-.08	-.01	.17	**.64**
	I become unable to concentrate when I recall the event	1.05	.91		.04	.16	.03	**.61**
Inter-factor correlations				Factor 1	-			
				Factor 2	.40	-		
				Factor 3	.22	.68	-	
				Factor 4	.42	.71	.64	**-**

### Convergent validity: ERPD and IES-R

As for the construct validities of the ERPD, feelings of revenge, rumination, self-denial, and mental paralysis had significant positive relationships with the IES-R subscales (*r*_s_ = 0.33–0.61; Table 4). The ERPD was significantly (and closely) correlated with other established indicators of PTSD-like symptoms, providing high support for the convergent validity of the ERPD. Both the scores of ERPD scale and the IES-R, however, were positively correlated with the severity of depressive symptoms (*r* = .41, *p* < .001; *r* = .50, *p* < .001, respectively). Therefore, we performed partial correlation coefficients between the ERPD scale and IES-R, after adjusting for sex, the period from the event occurrence, and QIDS-J scores. Although we found that correlations between ERPD and IES-R subscales were typically moderate, feelings of revenge (ranged .34–.40) and self-denial (ranged .21–.31) had lower correlations with the total IES-R and all its factors. This result shows the differentiation between the two scales.

**Table 4:** Pearson’s correlation and partial correlation analyses between the ERPD scale and the IES-R.

			ERPD scale			
		Total score	Factor 1: Feelings of revenge	Factor 2: Rumination	Factor 3: Self-denial	Factor 4: Mental paralysis
IES-R						
Zero-order correlation coefficients						
	Total score	.66	.45	.61	.41	.61
	Intrusion	.63	.41	.61	.38	.59
	Avoidance	.55	.39	.50	.37	.45
	Hyperarousal	.58	.41	.51	.33	.59
Partial correlation coefficients						
	Total score	.58	.40	.54	.31	.51
	Intrusion	.54	.35	.54	.29	.49
	Avoidance	.47	.34	.42	.29	.35
	Hyperarousal	.47	.35	.42	.21	.48

*Note*. Partial correlation coefficient between the ERPD scale and IES-R was adjusted for sex, the period from the event occurrence, and QIDS-J scores. ERPD, event-related psychological distress; IES-R, Impact of Event Scale-Revised; QIDS-J, Quick Inventory of Depressive Symptomatology; All correlations were significant (*p* < .05).

## Discussion

The purpose of this study was to determine the psychological features of ERPD after individuals experienced non-traumatic stressful life events, and to develop and validate the ERPD assessment tool through the implementation of the present qualitative and quantitative studies. This study yielded the identification that the psychological features of ERPD comprised four factors: feelings of revenge, rumination, self-denial, and mental paralysis, through the development of the ERPD-24, a novel self-report questionnaire, to assess ERPD in people distressed by their experiences of non-traumatic stressful events.

To our knowledge, this study was the first to identify psychological phenomena and features of ERPD after non-traumatic stressful events. The psychological features of ERPD consist of some similarities and differences with those of PTSD (Table 3). Regarding similarities, ERPD includes rumination and mental paralysis, which resembles the features of intrusive distressing memories, flashbacks, poor concentration, and physical/psychological distressing reactions to reminders of the stressful events in PTSD. Further, self-denial in ERPD resembles the negative alternations to cognitions and mood associated with PTSD, such as exaggerated self-blame and excessively negative thoughts and assumptions about one’s self [17]. The present findings confirmed that people with ERPD could have these PTSD-like symptoms.

Concerning differences, the feelings of revenge in the ERPD was the most different from PTSD because there is only one item in the diagnostic criteria for PTSD that resembles revenge: persistent, distorted cognitions about the cause or consequences of the traumatic events, leading the individual to blame himself/herself or others [17]. This indicates a key difference between non-traumatic and traumatic events. Trauma in PTSD and complex PTSD is associated with extremely life-threatening events including war, crime, natural disasters, terrible accidents, torture, refugees, childhood abuse, domestic violence, torture, and imprisonment [17, 24, 25]. In contrast, non-traumatic stressful events do not fall into these categories for a diagnosis of PTSD or complex PTSD (e.g., interpersonal problems such as discord between intimate persons, health problems, and individual economic crisis).

Approximately 65% of participants in this web-based survey answered that their non-traumatic but distressing events involved interpersonal problems. Forbes and colleagues reported that people who experience intimate interpersonal trauma are more likely to experience core symptoms of PTSD than those with non-intimate interpersonal or non-interpersonal trauma [26]. Moreover, compared with traumatic events, non-traumatic events are considered less threatening and more frequently appear in daily interpersonal activities (e.g., intimate person’s betrayal, discord among family members or friends, etc.). Given that, this finding suggests that people with ERPD owing to interpersonal problems could be likely to harbor feelings of revenge, hate, anger, and blame against the other persons causing distress. Therefore, when therapists care or treat people with ERPD owing to interpersonal problems, they should evaluate their feelings of revenge against the causes to understand the state of mind of those people.

Regarding the other difference from PTSD, in this study, avoidance and avoidance-related features were not included in the content of the ERPD-24. Avoidance is required in the diagnostic criteria C for PTSD [17]. Several reasons were considered to explain this difference. One major reason is that non-traumatic events are not life-threatening. A second reason is that such events (e.g., economic problems and health problems) are not necessarily something that can be avoided, although traumatic events must be avoided for people to save their lives. However, interpersonal problems associated with ERPD are complex and materially vary per person. A further reason is that some people with ERPD experience feelings of revenge against a person, people, or various groups (e.g., classmates, co-workers) who are etched in their mind as being connected to unpleasant memories leading to their ERPD. In cases in which “feelings of revenge” are more predominant than other factors in EPRD, those people may be unlikely to have avoidance and avoidance-related features in their ERPD. Therefore, as a core symptom of ERPD, avoidance may not necessarily be an essential factor of ERPD differing from that of PTSD. Further studies are needed to investigate the relationship between ERPD and types of related events.

This study also demonstrated that the severity of depressive symptoms was positively correlated with that of ERPD through the web-based survey. This finding is consistent with that of our previous studies for patients with mood disorders [11, 12]. ERPD phenomena are induced and affected by distressing episodic memories. Experimental studies regarding the relationship between memory and mood (or emotion) can help clarify ERPD and depressed mood. Lloyd and Lishman reported that patients with more severe depression take longer to recall pleasant experiences as opposed to unpleasant experiences [27]. Furthermore, Buchanan [28] and Bower [29] showed that people in a sad (or happy) mood are more likely to retrieve sad (or happy) events, and that depressed individuals prolong their depressed mood by recalling unpleasant events. Given that exposure to non-traumatic stressful events causes severe mood disturbances in some people, and that it can trigger the onset and relapse of mood disorders [4, 6–8], further longitudinal studies are required to clarify the relationships between mood changes in patients with ERPD and those with other mood disorders or mental illnesses prone to depression. This could help clarify the management of mental illnesses.

Previous studies used conventional instruments (e.g., the IES-R) to assess the severity and psychometric profiles of ERPD- or PTSD-like symptoms associated with non-traumatic events. However, the current ERPD-24 is useful to assess the severity of ERPD associated with non-traumatic life events. In validating the ERPD-24 against the IES-R, it became clear that feelings of revenge feelings and self-denial could be unique features of ERPD.

The ERPD-24 can help therapists understand, assess, and treat people with ERPD associated with non-traumatic events, which may differ from those diagnosed with PTSD. Moreover, the ERPD-24 may be applied to not only people with mood disorders but also those with anxiety disorders, developmental disorders, or subclinical people with depressive symptoms. Previous studies reported that, after stressful life events, patients with anxiety disorders such as generalized anxiety disorder or panic disorder were at an increased risk of relapse [30, 31]. In addition, patients with autism spectrum disorders tend to experience stressful life events and feel stress [32]. Furthermore, these psychiatric disorders are highly comorbid with depression [33, 34].

Additionally, in Step 2 of this study, approximately 90% of the web-based general population answered that they did not have ERPD. The other 10% of those were judged to have moderate or higher depressive symptoms according to their QIDS-J scores [22, 23]; although, we excluded people with major psychiatric disorders including depression at the screening stage. However, the rate of depressive symptoms in the web-based survey may be close to that of the general population, since Kroenke and colleagues reported that 8.6–9.1% of the general population have depressive symptoms [35]. Given that, the ERPD-24 is also useful to manage patients with anxiety or developmental disorders, as well as those with subclinical depressive symptoms who have ERPD associated with non-traumatic stressful events.

This study had several limitations. First, all participants were from Japan; therefore, our results cannot be generalized to other cultures. Regarding cultural differences associated with personality traits, Eap and her colleagues showed that the personality of Asian Americans tends to be more introverted and neurotic than that of European Americans [36]. In particular, several studies reported that the Japanese tend to be more self-critical than Westerners [37, 38]. Thus, the perception and attitude to stressful life events of Japanese and/or Asian people may be different from those of Westerners if they experience the same events. Hence, the contents of ERPD may also vary based on race-specific cultures. Furthermore, concerning the cultural and security situations of countries or regions, various recognitions of non-traumatic events are considered to depend on the countries and their regions due to the differences in security, political stability, and economics. Here, it is noteworthy that even in developed countries, the aforementioned issues exist. For instance, according to the white paper from the Ministry of Justice of Japan, the rates of homicide in 2017 was 0.2 per 100,000 in Japan, which was considerably lower than 1.2 in the United Kingdom, 1.3 in France, and 5.3 in the United States [39]. Therefore, non-traumatic life events that people consider to be stressful may also depend on the degree of security in each country or region. Although we conducted this study and developed the ERPD-24 in Japanese, to generalize the concept of ERPD, international collaborative studies are needed.

Second, the range of difference in the use of diagnostic criteria A for PTSD between trauma and non-traumatic events are considered reasonably large worldwide [17]. Moreover, since we assessed non-traumatic events only, the severity of the events may also largely differ among each person. Recent studies reported that PTSD-like symptoms were observed in people distressed by interpersonal problems including various kinds of harassment (e.g., power and sexual harassment at school [40] and the workplace [41]) and school bullying issues among children [42]. Even if these stressful events do not objectively meet the traumatic ones required for diagnosing PTSD, these stressful events and the related distressed conditions are serious. According to the current diagnostic criteria for PTSD and complex PTSD in the DSM-5 and ICD-11 [25], these serious events are thought to be excluded from traumatic events. If so, the concept of ERPD might be meaningful for people distressed with psychological symptoms after non-traumatic stressful events. Although this novel study helps elucidate the concept of ERPD and provides an initial assessment tool of ERPD, further studies to refine those are needed.

In conclusion, we clarified the psychological features of ERPD in people distressed by non-traumatic stressful experiences, and we developed a novel self-report assessment of ERPD, which was independent from that of PTSD. For various professionals involved in mental healthcare, this tool can help stakeholders understand and assess psychological phenomena in people with ERPD. Further studies are needed to apply this ERPD assessment tool to people with distinct mental health problems and illnesses.
